# *Drosophila* Rbp6 Is an Orthologue of Vertebrate Msi-1 and Msi-2, but Does Not Function Redundantly with *d*Msi to Regulate Germline Stem Cell Behaviour

**DOI:** 10.1371/journal.pone.0049810

**Published:** 2012-11-27

**Authors:** Nicole A. Siddall, Marina Kalcina, Timothy M. Johanson, Adrian C. Monk, Franca Casagranda, Reeva P. Been, Eileen A. McLaughlin, Gary R. Hime

**Affiliations:** 1 Anatomy and Neuroscience, University of Melbourne, Parkville, Australia; 2 ARC Centre of Excellence in Biotechnology and Development, Callaghan, Australia; 3 School of Environmental and Life Science, University of Newcastle, Callaghan, Australia; University College London, United Kingdom

## Abstract

The vertebrate RNA-binding proteins, Musashi-1 (Msi-1) and Musashi-2 (Msi-2) are expressed in multiple stem cell populations. A role for Musashi proteins in preventing stem cell differentiation has been suggested from genetic analysis of the *Drosophila* family member, *d*Msi, and both vertebrate Msi proteins function co-operatively to regulate neural stem cell behaviour. Here we have identified a second *Drosophila* Msi family member, Rbp6, which shares more amino acid identity with vertebrate Msi-1 and Msi-2 than *d*Msi. We generated an antibody that detects most Rbp6 splice isoforms and show that Rbp6 is expressed in multiple tissues throughout development. However, *Rbp6* deletion mutants generated in this study are viable and fertile, and show only minor defects. We used *Drosophila* spermatogonial germline stem cells (GSC’s) as a model to test whether *Drosophila* Msi proteins function redundantly to regulate stem cell behaviour. However, like vertebrate Msi-1 and Msi-2, Rbp6 and Msi do not appear to be co-expressed in spermatogenic GSC’s and do not function co-operatively in the regulation of GSC maintenance. Thus while two Msi family members are present in *Drosophila*, the function of the family members have diverged.

## Introduction

Stem cells are characterised by their ability to both self-renew, and to produce a daughter cell fated for differentiation. Genes that are required to regulate the balance between stem cell self-renewal and differentiation are gradually being identified [1], [2], [3], [4], and an understanding of the regulation of these stem cell factors is essential to enhance the potential of using these factors as therapeutic targets in stem cell based therapies. The Musashi (Msi) family of RNA-binding proteins have been shown to be expressed in a number of different stem cell populations and are thought to be integral to the regulation of stem cell behaviour [5].

The first member of the Msi family (*d*Msi; CG5099) was identified in *Drosophila*, and was shown to play an essential role in the regulation of the asymmetric division of sensory organ precursor cells (SOPs), the multipotent progenitor cells that give rise to external sensory organs [6], [7]. *d*Msi was shown to prevent translation of the neural differentiation inhibitory factor, Tramtrack69 (Ttk69), specifically in neural precursor cells of the SOP lineage by binding to consensus sequences in the 3′ UTR region of *ttk69* mRNA [7]. *d*Msi is also required to negatively regulate Ttk69 in the developing *Drosophila* eye [8], [9]. Our laboratory has since shown that *d*Msi is also required to regulate spermatogonial stem cell maintenance by preventing the premature differentiation of stem cells via a novel, unidentified mechanism [10].

**Figure 1:**
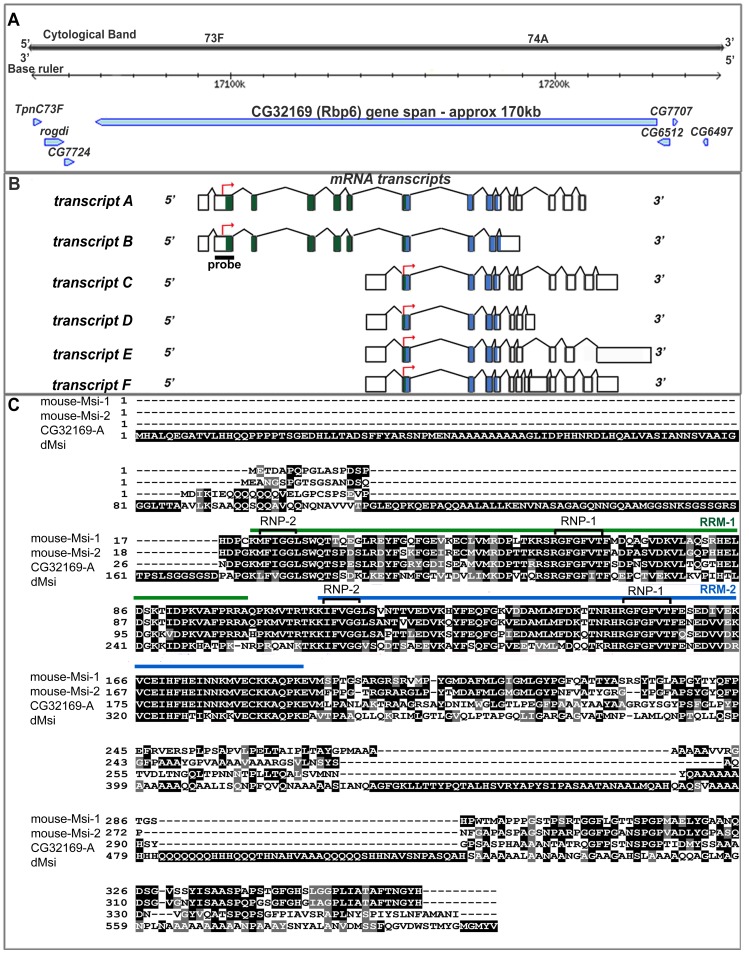
CG32169, also known as Rbp6, is a paralogue of dMsi and an orthologue of mammalian Msi proteins. (A) Genomic representation of CG32169 (Rbp6) on chromosome 3L (modified from Flybase). (B) CG32169 (Rbp6) has six transcripts. Exons encoding for RRM1 and RRM2 are coloured green and blue respectively, and coding start sites are indicated (red arrow). The region to which the RNA probe was designed is underlined. (C) Sequence alignment of CG32169 (Rbp6) isoform A with *d*Msi, mouse Msi-1 and Msi-2 using CLUSTALW. Single-letter amino acid codes are used. Alignments among the four proteins are highlighted by black boxes for identical amino acids, and by grey boxes for similar amino acids. The two RNA recognition motifs are noted with a green or blue line. Each RRM includes two highly conserved sequences designated RNP-1 and RNP-2.

Two Msi orthologues, Msi-1 and Msi-2, were later identified in vertebrates. Evolutionary analysis has shown that these proteins arose by gene duplication and are conserved in most vertebrate species, including chick, mouse, human and dog [11]. Expression of both Msi1 and Msi2 has been observed in mouse neural stem/progenitor cells in the ventricular and subventricular zone throughout central nervous system (CNS) development, leading to the hypothesis that Msi proteins may play an important role in neural stem cell (NSC) regulation [12], [13], [14]. The expression of Msi-1 and Msi-2, although overlapping in NSC/progenitor cell populations of the CNS, differ in that Msi-1 expression is rapidly down-regulated in post-mitotic neurons in the cerebral cortex, while Msi-2 is continuously expressed in a subset of some GABAergic interneurons [14]. This shows that the two proteins have, to some extent, functionally diverged. Nevertheless, functional analysis of Msi-1 and Msi-2 in mammalian NSC regulation has revealed the co-operative nature of these two proteins in maintaining the undifferentiated state of NSCs, since disruption of either Msi-1 or Msi-2 alone showed no effect on the number or self-renewing activity of NSCs, but disruption of Msi-1 and Msi-2 together resulted in the sharp reduction of neurosphere formation due to the reduced self-renewal potential of NSCs [15]. This and subsequent molecular experiments have led to the conclusion that Msi-1 and Msi-2 have important functions in maintaining NSC fate through translational repression of *numb* mRNA, allowing for the activation of Notch (N) signalling which, in turn, positively regulates NSC self-renewal [15], [16]. Both mouse Msi-1 and Msi-2 contain two sets of ribonucleoprotein (RNP)-type RNA recognition motifs (RRMs), with the RRM regions of Msi-2 sharing 85% amino acid identity with Msi-1, supporting the idea that Msi may share the same or similar target RNAs and compensate for each other’s function [14].

**Figure 2:**
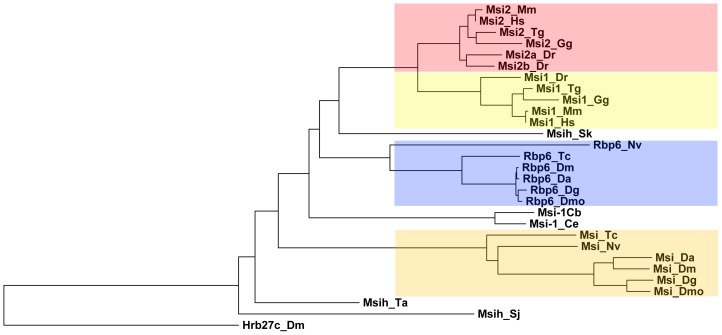
Phylogram of Msi family sequences. Vertebrates contain two Msi paralogues, Msi2 (red box) and Msi1 (yellow box). A single orthologue found in the hemichordate (Msih_Sk) is closely aligned with the vertebrate sequences. The Rbp6 clade (blue box) of Msi proteins is insect-specific but also more closely aligned with the vertebrate proteins than the single orthologues found in nematodes. The Msi clade (orange box) is a more highly derived insect-specific group of proteins. Single representatives of the Msi family can be found in Trichoplax and Schistosoma. The RRM-containing protein, Hrb27c, was used as an outgroup. Mm: *Mus musculus* (mouse), Hs: *Homo sapiens* (human), Gg: *Gallus gallus* (chicken), Tg: *Taeniopygia guttata* (zebra finch), Dr: *Danio rerio* (zebra fish), Sk: *Saccoglossus kowalevskii* (acorn worm/hemichordate), Nv: *Nasonia vitripennis* (jewel wasp), Tc: *Tribolium castaneum* (red flour beetle), Dm: *Drosophila melanogaster* (fruit fly), Da: *Drosophila ananassae* (fruit fly), Dg: *Drosophila grimshawi* (fruit fly), Dmo: *Drosophila mojavensis* (fruit fly), Ce: *Caenorhabditis elegans* (nematode), Cb: *Caenorhabditis briggsiae* (nematode), Ta: *Trichoplax adhaerens* (placozoan), Sj: *Schistosoma japonicum* (blood fluke).

The restricted pattern of Msi-1 expression in neural stem/precursor cell populations suggested that Msi-1 primarily functions in undifferentiated cells, leading to the proposal that Msi-1 is a stem cell marker. Indeed, Msi-1 is expressed in approximately 30–50 cells in the mouse intestinal crypt, which encompass intestinal stem cells and transit-amplifying cells [17], [18], [19]. Expression of Msi-1 has also been detected in mouse spermatogonial stem cells and in epithelial progenitors of the mammary gland, hair follicle, epidermis and human antrum [10], [20], [21], [22]. In addition, an increase in Msi-1 expression has been observed in some colorectal cancers [23], in brain tumours [24], [25] and in endometrial carcinomas [26], all of which may have a stem cell origin. Mouse studies have also recently shown that adenomas in the small intestine are derived from intestinal stem cells, a Msi-1 positive cell population [27]. Thus Msi-1 presents itself as a potential therapeutic target in anti-cancer therapy. Recent advances have also been made towards elucidating the role of Msi-2 in regulating stem cell behaviour. Msi-2 is essential for the maintenance of the primitive fate of hematopoietic cells through governing the balance between stem cell self-renewal and differentiation [28]. Very recently, Msi-2 has been shown to be required for the self-renewal of embryonic stem cells [29]. Additionally, Msi-2 is expressed in the region where mouse hair follicle precursor cells lie, and Msi-2 co-operatively functions with Msi-1 in the regulation of NSC behaviour [15], [22].

The Msi family has an ancient origin in the history of metazoans. A single Msi orthologue is present in the genomes of the sequenced nematodes, *Caenorhabditis elegans* and *Caenorhabditis briggsiae*, and representatives can be identified in the platyhelminth, *Schistosoma japonicum*, as well as the primitive placozoan, *Trichoplax adhaerens*, which possibly represents one of the simplest free-living animals. To date, only one Msi protein has been identified in *Drosophila melanogaster*. Consistent with the hypothesis that Msi is functionally required in stem cell populations, our previous study showed that *d*Msi is intrinsically required in spermatogonial GSCs for maintenance of GSC identity [10]. A revised annotation of the *Drosophila* genomic sequence suggested that a Msi paralogue is present in *Drosophila melanogaster.* We show by phylogenetic analysis and protein alignment that RNA-binding protein 6 (Rbp6), also known as CG32169, shares more sequence identity with the vertebrate Msi-1 and Msi-2 proteins than its *Drosophila* paralogue, and that *d*Msi subsequently arose from a gene duplication event within the insect lineage. We show by *in situ* hybridisation and antibody analysis that Rbp6 is expressed in the photoreceptor cells of third instar eye discs, the third instar ring gland and brain lobes, the somatic cells of the adult testis and in the oocyte of the ovary.

**Table 1:** Intron and Exon sizes of Rbp6 splice isoform A.

	Exon	Intron
**1**	379 bp	1,769 bp
**2**	711 bp	13,509 bp
**3**	41b p	42,426 bp
**4**	82b p	12,194 bp
**5**	85 bp	2,060 bp
**6**	42 bp	35,941 bp
**7**	93 bp	44,722 bp
**8**	49 bp	5,909 bp
**9**	83 bp	409 bp
**10**	85 bp	3,233 bp
**11**	36 bp	76 bp
**12**	84 bp	6,134 bp
**13**	66 bp	624 bp
**14**	242 bp	296 bp
**15**	93 bp	

We generated deletions covering different regions of the *Rbp6* locus, but surprisingly, the *Rbp6* deletion mutants were viable and fertile, and exhibited no obvious external adult morphological defects other than homozygous mutant flies were slower to eclose than their balanced heterozygote counterparts. Additionally, we show that Rbp6 is not required to maintain the fate of germline stem cells, and plays no role in male meiotic processes, unlike its paralogue *d*Msi. The only testis phenotype we did observe in mutants was the occasional mislocalisation of the testis niche. The analysis of *Rbp6/msi* double mutant recombinants showed that these genes do not function redundantly to regulate the maintenance of spermatogonial stem cells, as no enhancement of the *msi* mutant phenotype was observed in the double mutants. However, forced mis-expression of Rbp6 in the early germline caused a loss of germ cells due to cell death, suggesting that Rbp6 levels must be tightly regulated to prevent apoptosis of cells, and suggests some function for the gene. Thus while vertebrate Musashi family members may functionally co-operate in at least some developmental processes, the invertebrate Musashi proteins, *d*Msi and Rbp6, have functionally diverged such that there appears to be no functional redundancy between the two proteins.

**Table 2:** Percentage amino acid identity between Mouse and *Drosophila* Musashi family proteins.

	Mouse Msi-1	Mouse Msi-2	*Drosophila* Msi
**Rbp6-A**	**49%**	**54%**	**36%**
**Rbp6-B**	**37%**	**33%**	**30%**
***Drosophila*** ** Msi**	**32%**	**39%**	

## Materials and Methods

### Fly Strains

The fly stocks used in this study include *w^1118^, msi^1^* and *msi^2^* [6], nanosGal4VP16 (*nos*Gal4) [30], P{hsFLP}12;TM3/TM6B, TM3/TM6B, and Df(3L)81k19/TM6B, all of which were obtained from the Bloomington Stock Center. The fly stocks used to generate the *Rbp6^1^, Rbp6^2^ and Rbp6^3^* deletions, f07212, d06909, d11241, f03754, f10793 and d10465 were obtained from the Exelexis stock collection [31]. All flies were raised on standard molasses-based food at 25°C except for Gal4 crosses, which were all conducted at 29°C.

**Figure 3:**
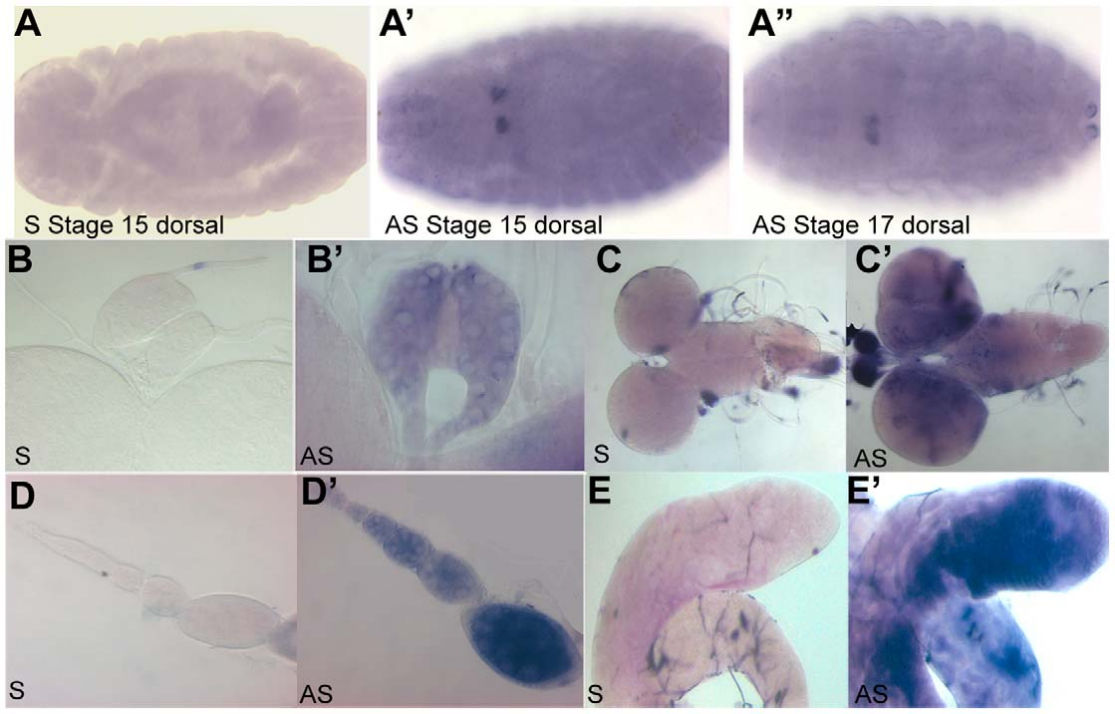
Expression of *Rbp6* mRNA throughout development. (A–A’’) *Rbp6* mRNA is detected in the prothoracic gland precursor cells in the embryo. *Rbp6* mRNA is also detected in the third instar prothoracic gland (B–B’), the third instar larval brain (C–C’), the ovarioles of the adult ovary (D–D’) and in the adult testis (E–E’). S-Sense, A-Antisense.

### Generation of Rbp6 Deletion Lines

For the generation of *Rbp6^1^* mutants, P{hsFLP}12;TM3/TM6B virgin females were mated with f10793 males carrying a WH piggyBac element. Males carrying both the hsFlp and the WH element were then crossed to d10465 virgin females carrying an XP element. After both 48 hours and 72 hours, the progeny were heat shocked for 1 hour at 37°C in a water bath. The progeny were raised to adulthood and virgin females were then mated to a *w1118*; TM3/TM6B balancer stock. The generated mutant could be selected for on the basis of a change in eye colour from red eyes to white eyes [31], thus 5 white eyed individuals were chosen to generate stocks from. The deletion was then confirmed by PCR. For the generation of *Rbp6^2^* mutants, the same protocol was repeated with lines d11241 (XP element), and f03754 (WH element). *Rbp6^2^* mutants could also be selected for on the basis of eye colour change and were confirmed by PCR [31]. For the generation of *Rbp6^3^* mutants, a similar crossing scheme was instigated with lines f07212 (WH element) and d06909 (XP element), only *Rbp6^3^* mutants could not be selected for on the basis of a change in eye colour. In this case, 50 balanced stocks were generated using the crossing scheme described above, and mutants were identified by PCR. Mutants were also confirmed by analysis with the Rbp6 antibody. For further information on construction of deletions using DrosDel P elements, please visit http://www.drosdel.org.uk/# or refer to [31].

**Figure 4:**
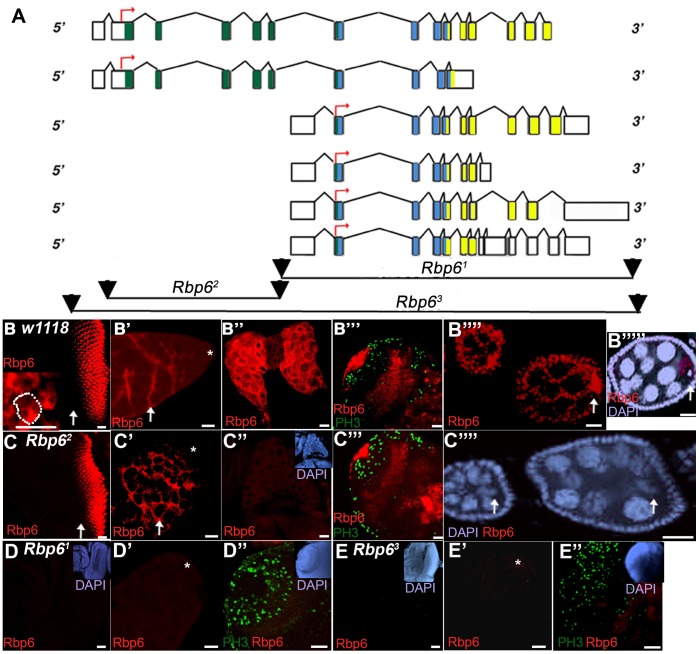
Expression of Rbp6 protein throughout development in *w^1118^* and *Rbp6* mutant tissue. (A) Schematic of *Rbp6* transcripts. The peptide synthesized for the generation of the Rbp6 antibody was derived from the exonic sequences depicted in yellow, thus the possibility remains that splice isoforms A, C, D, E and F could be detected with this antibody. The deletions generated in our study span different regions of Rbp6 (depicted at the bottom of A). (B) Rbp6 expression (red) in tissue dissected from *w^1118^* flies shows that Rbp6 is expressed posterior to the morphogenetic furrow (arrow) in third instar larval eye discs (B), in the cytoplasm of somatic cyst cells (arrow) in the adult testis (B’; hub is denoted by *), in the cytoplasm of cells in the third instar ring gland (B’’), in the non-proliferative cells of the third instar brain lobe (B’’’; dividing cells are labelled with Phosphohistone H3 (green)), and in the oocyte and nurse cells of the adult ovary (arrow; B’’’’–B’’’’’). In *Rbp6^2^* mutant tissue, no antibody expression was detected in the third instar ring gland (C’’) or the oocyte of the ovary (arrow; C’’’’), but was detectable in the eye disc (C), adult testis (C’) and larval brain lobe (C’’’). Rbp6 antibody expression (red) was undetectable in both Rbp6*^1^* (D–D’’) and *Rbp6^3^* (E–E’’) mutant tissue. Scale bars: 10 µm.

### Generation of the *UAS-Rbp6* Line

For the generation of *UAS-Rbp6*, the entire coding region of Rbp6 isoform A was amplified by PCR from a cDNA clone (RE25373) template obtained from the *Drosophila* Genomics Resource Center (DGRC). The PCR product was then subcloned into an Invitrogen™ pCR®II-TOPO® TA cloning vector according to manufacturers instructions. The insert was digested with *Xba*I and *Bgl*II and directionally cloned into a pUAST vector [32]. This construct was then transformed into a *w^1118^* host strain using standard methods to generate transgenic lines [33].

### Generation of the *msi^1^Rbp6^1^* and *msi^2^Rbp6^1^* Recombinant Lines

For the generation of recombinant lines, balanced stocks were created from *msi^1^*/*Rbp6^1^* and *msi^2^/Rbp6^1^* female parents crossed to a third chromosomal balancer stock. Each individual stock was assayed for the *msi* mutant external bristle phenotype [6]. Genomic preparations were obtained from these flies and the presence of a deletion in the *Rbp6* region was confirmed by PCR.

**Figure 5:**
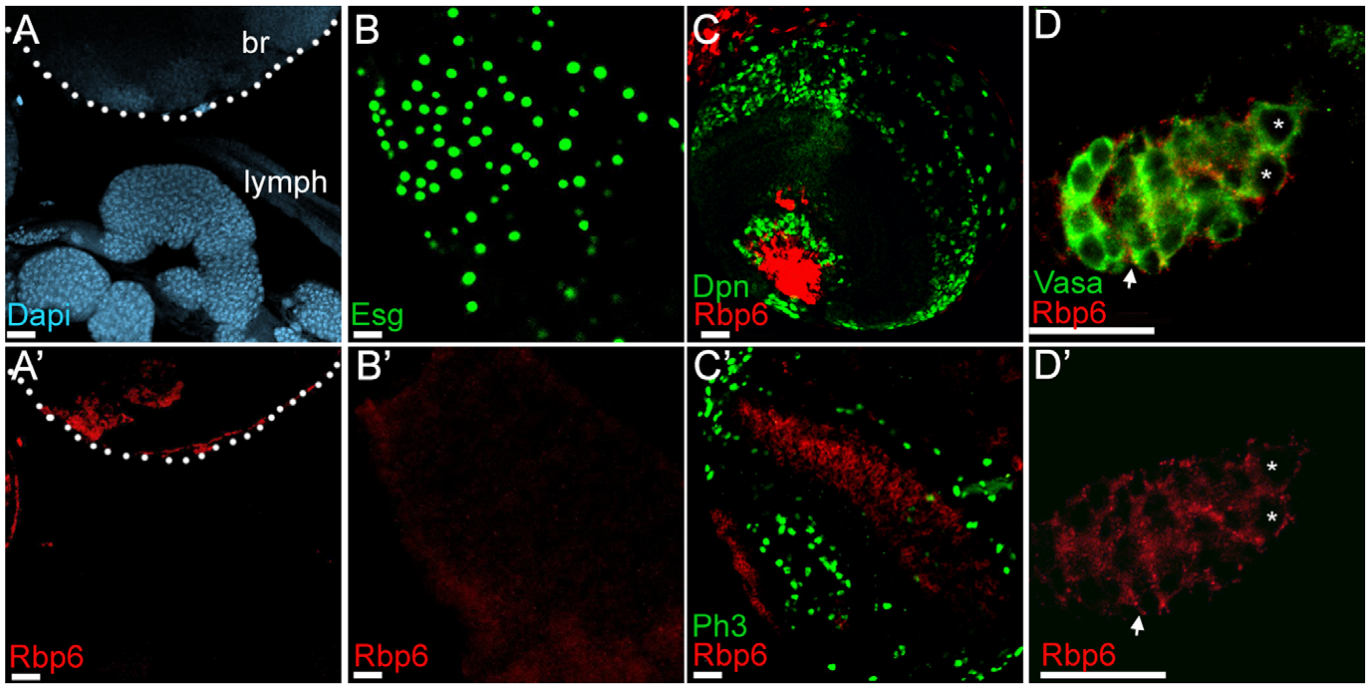
Rbp6 is not widely expressed in stem cell populations at different developmental stages. Rbp6 antibody staining (red in all panels) was not detected in the lymph gland (A–A’), the source of hematopoietic stem cells, in intestinal stem cells (which are identified by the expression of Escargot (green, B–B’), or in proliferating neuroblasts of the third instar larval brain, which are marked by either Deadpan expression (green, C) or phospho-histone H3 expression (green, C’). Rbp6 expression was detected at low levels in the germline stem cells (asterix) and possibly the somatic stem cells (arrow) of the adult ovary (D–D’). Scale bars: 20 µm. br represents the brain lobe (A–A’).

### PCR Confirmation of Deletion Mutants

The PCR confirmation of *Rbp6^1^*, *Rbp6^2^* and *Rbp6^3^* mutations generated in this study were carried out as described in the supplementary methods of [31]. A hybrid PCR was performed for the confirmation of the *Rbp6^1^* and *Rbp6^2^* deletions using primers specific for the hybrid P-element generated as a result of the deletion events. These primers are described in [31]. In each of these cases, a resultant 1.8 kb PCR product would confirm that a deletion event had occurred. For the confirmation of the *Rbp6^3^* deletion, a two-sided PCR was carried out using WH3’minus and XP3’plus primers described in [31] in combination with the following Rbp6 specific genomic primers: 5′-CTG CCC TCT TTG ATG TCC TG-3′ and 5′-GAC CTG TCA GTG TGG AAA AGC-3′ respectively. A successful deletion would result in the amplification of 1.2 kb and 400 bp PCR products from flies carrying the full length deletion.

### Generation of the Rat Polyclonal Rbp6 Antibody

To generate the rat Rbp6 polyclonal antibody, the C-terminal portion of Rbp6 isoform A was amplified using the following primers: [5′-CAC CAA TCT GGC CCA AGA CGC-3′] and [5′-TTA TAT ATT AGC CAT CGC AAA GTT C-3′]. The PCR product was cloned into a pENTR™/D-TOPO® vector and then was subsequently cloned into a Gateway® pDEST™15 vector containing a GST tag according to manufacturer’s instructions (Life Technologies). The integrity of the PCR fragment was sequence verified. The segment was bacterially expressed as glutathione *S*-transferase fusion protein (GST-Rbp6) and the antigen sent in a gel for production of a rat polyclonal antibody to the Institute of Medical and Veterinary Science (IMVS, Adelaide, Australia).

### Affinity Purification of Rbp6 Polyclonal Antibody

GST-Rbp6 protein (10–100 µg) was run on an acrylamide gel and transferred onto an Immobilon-P membrane. The membrane was stained with Ponceau Red for 2 minutes, then washed quickly with water until protein bands became visible. The GST-Rbp6 protein band was cut out, blocked in 5% milk powder, washed and incubated with anti-Rbp6 antisera for 1 hour at RT. The membrane strip was washed in PBS, and stripped in 300 uL 5 mM glycine, 150 mM NaCl pH 2.4 for 30 minutes at RT. The supernatant was neutralized by the addition of 100 uL Na_2_PO_4_ pH 8. The affinity purified antisera was stored at 4°C.

### RNA *in situ* Hybridisation

For generation of *Rbp6* antisense and sense probes, PCR products were amplified from a cDNA clone template (RE25373), which was obtained from the Drosophila Genomics Resource Center (DGRC). To generate an antisense probe, the forward primer containing the T7 polymerase sequence, 5′-TGTAATACGACTCACTATAGGGGCTGGAGCTGGTGATTGGAG-3′, was used in conjunction with the reverse primer 5′-CTAGAGCAGGAGAAAAGCCGTG-3′. To generate a sense probe, the forward primer, 5′-TGTAATACGACTCACTATAGGGCTAGAGCAGGAGAAAAGCCGT-3′, which contained the T7 polymerase sequence, was used in conjunction with the reverse primer sequence, 5′-GCTGGAGCTGGTGATTGGAG-3′. The 361-bp products were then gel purified using a Qiaquick gel extraction kit according to manufacturer’s instructions. Digoxygenin-labelled antisense and sense RNA probes were generated using the Ambion T7 Megascript® Kit with Roche Dig-NTP labelling mix according to manufacturer’s instructions. The RNA *in situ* hybridisation protocol was carried out as previously described [34].

### *Drosophila* Immunohistochemistry, Acridine Orange Staining, Phase Microscopy and Confocal Microscopy

*Drosophila* tissue was dissected and stained as described [35]. Antibodies were used at the following noted dilutions: 1∶50 rat anti-Rbp6, 1∶5000, rabbit anti Phosphohistone H3 (Millipore), 1∶50 mouse anti-FasciclinIII (FasIII; Developmental Studies Hybridoma, DSHB), 1∶100 goat anti-Vasa (Santa Cruz Biotechnology), 1∶10 rat anti-Musashi (H. Okano), 1∶20 rat anti-DE-Cadherin (Dcad2; DSHB), 1∶20 mouse anti-1B1 (DSHB), 1∶5000 rabbit anti-Zfh1 (gift of R. Lehmann), 1∶500 guinea-pig anti-Deadpan (gift of J Skeath), 1∶500 guinea-pig anti-Numb (gift of M. Murray). Diamidino-2-phenylindole (DAPI) was present in the mounting reagent, Prolong® Gold (Molecular Probes, Invitrogen). All secondary antibodies were Alexa Fluor antibodies obtained from Molecular Probes. Serial confocal sections were imaged on a Zeiss LSM510 Confocal Microscope. Phase microscopy imaging of meiotic spermatids from live testis preparations was carried out as previously described [10]. Acridine orange staining was performed on 0–3 day old adult testes (post eclosion) raised at 29°C according to [36].

### *Drosophila* Developmental Delay Assay

100 *Rbp6^1^/Df(3L)81k19* or *Rbp6^3^/Df(3L)81k19* mutant 1^st^ instar larvae and 100 *Rbp6^1^*/TM3-GFP(Ser) or *Rbp6^3^*/TM3-GFP(Ser) 1^st^ instar larvae were selected for by the presence or absence of GFP and placed in a vial of food. The numbers of eclosed adults were recorded from 11 days post egg deposition.

### Bioinformatics and Statistical Analysis

To create the phylogenetic tree, protein sequences were aligned using ClustalW2 (http://www.ebi.ac.uk/Tools/clustalw2/index.html) and the tree constructed using TreeCon (http://bioinformatics.psb.ugent.be/software/details/3). Protein sequences were accessed at (http://www.ncbi.nlm.nih.gov/protein). Sequence accessions are: Msi2_Mm, NP_473384; Msi2_Hs, NP_620412; Msi2_Tg, XP_002196910; Msi2_Gg, XP_415912; Msi2a_Dr, NP_997961; Msi2b_Dr, NP_957403; Msi1_Dr, NP_001013534; Msi1_Tg, XP_002196403; Msi1_Gg, XP_415271; Msi1_Mm, NP_032655; Msi1_Hs, NP_002433; Msih_Sk, NP_001158364; Rbp6_Nv, XP_001606007; Rbp6_Tc, XP_968171; Rbp6_Dm, NP_730245; Rbp6_Da, XP_001956784; Rbp6_Dg, XP_001984346; Rbp6_Dmo, XP_002007249; Msi-1Cb, XP_002647098; Msi-1_Ce, NP_497799; Msi_Tc, XP_968418; Msi_Nv, XP_001607438; Msi_Da, XP_001953309; Msi_Dm, NP_733108; Msi_Dg, XP_001989774; Msi_Dmo, XP_002000660; Msih_Ta, XP_002115015; Msih_Sj, CAX72901; Hrb27c_Dm, NP_476869.

To calculate the amino acid identity between proteins, the Needleman-Wunsch global pairwise alignment algorithm method was used, which is available online from the European Bioinformatics Institute. To calculate the significance values for the ovary mosaic analysis, a standard Chi Square test was performed. To calculate the significance values and standard error of the mean for stem cell counts in testes, Column Graph statistics and One-way ANOVA analysis was performed using GraphPad Prism software.

## Results

### CG32169 is a Paralogue of *d*Msi, and an Orthologue of Mammalian Msi Proteins

Since two vertebrate Musashi parologues have been shown to function redundantly to regulate NSC behaviour [15], we considered whether a parolog of *d*Msi was present in the *Drosophila* genome. Using a phylogenetic tree analysis program [37], we discovered that a RNA-binding protein related to *d*Msi did exist. CG32169, also known as Rbp6, spans 172,933 bp of chromosome 3L (Fig. 1A). Rbp6 comprises of six predicted annotated transcripts (Fig. 1B). Two long transcripts (A and B) encode for proteins containing two RNA Recognition Motifs (RRMs), while four shorter transcripts (C, D, E and F) encode for proteins containing only the second RRM (Fig. 1B). All isoforms demonstrated an unusual intron exon structure, with many very short exons of 85 bp or less neighbouring very large introns of up to 45 kb in size (Fig. 1B). Of the 15 exons of isoform A, 12 are less than 93 bp in size, while 7 of the 14 introns are over 5.5 kb in size, with 3 being over 30 kb in size (Table 1). Rbp6 isoform A (Rbp6-A) shares 36% amino acid (aa) identity with *d*Msi, while Rbp6-B shares 30% aa identity with *d*Msi (Table 2). Strikingly, when comparing the *Drosophila* Rbp6 and Msi protein sequences to the mouse Msi-1 and Msi-2 orthologues, Rbp6-A shares the most aa identity to both mouse Msi-1 and Msi-2 (49% and 54% respectively), with *d*Msi only sharing 39% aa identity to mouse Msi-2, and 36% identity to mouse Msi-1 (Table 2). Within the region of the RRM’s, Rbp6-A shares 78.7% aa identity with mouse Msi-1, and 82.2% identity with mouse Msi-2. In contrast, *d*Msi shares 58.7% aa identity with mouse Msi-1, and 61.1% aa with mouse Msi-2 in the region containing the RRMs. Additionally, the core RNP consensus sequences (RNP-1 and RNP-2) within each RRM, which are the domains necessary for binding RNA [38], are fully conserved between Rbp6-A and mouse Msi-1 and Msi-2, but are only partially conserved between *d*Msi and mouse Msi-1 and Msi-2 (Fig. 2C).

### Rbp6 is Representative of the Ancestral Msi Protein Sequence

While it was originally thought that *d*Msi was the only Msi-like sequence in *Drosophila*, it is clear that vertebrate and *Drosophila* genomes both contain two Msi paralogues, with the two proteins in both genomes having arisen from separate gene duplication events. The first identified member of the family, *d*Msi, is representative of an insect specific clade (orange box, Fig. 2) that has further evolved within the *Drosophilidae* from the acquisition of several sequence insertions (not shown). The Rbp6 clade (blue box, Fig. 2) is also unique to insects but it is more closely related to the vertebrate sequences (red and yellow boxes, Fig. 2) than the Msi clade. A single family member found in the hemichordate (*Saccoglossus kowalevskii*) is more similar to vertebrate Msi1 and Msi2 than the insect proteins, indicating that a duplication arose within chordate descendants to generate the two members observed in all vertebrates. A single Msi member is also present in *Ciona intestinalis* (although there is some confusion with the gene name, as by our analysis, *CiMsi* appears to be more similar to the DAZAP-1 family than Msi (not shown), again suggesting that the duplication event occurred post-chordate evolution. The single representatives present in the nematodes *C. elegans* and *C. briggsiae* are more similar to the Msi1/Msi2 and Rbp6 clades suggesting that the insect Msi clade represents a highly derived group that has diverged away from the core Msi family sequence.

### *In situ Hybridisation* Shows that *Rbp6* RNA is Expressed in Multiple Tissues throughout *Drosophila* Development

Because the Rbp6 long isofoms encoded two RRMs (Fig. 1B), a feature of all vertebrate Musashi proteins, we wanted to determine whether the *Rbp6* long transcripts were expressed throughout development. We performed RNA *in situ* hybridisation using a probe specifically designed to the second exon of the gene, which is shared by both Rbp6 splice isoforms A and B (Fig. 1B). *Rbp6* transcripts were detected in the ring gland precursor cells in embryos (Fig. 3A’–A’’), in the third instar ring gland (Fig. 3B’), in the third instar larval brain and ventral ganglion (Fig. 3C’) and in the adult ovary and testis (Fig. 3D’, E’). The wide-spread distribution of *Rbp6* expression throughout *Drosophila* development suggested to us that Rbp6 may be functionally required for a range of developmental processes.

**Figure 6:**
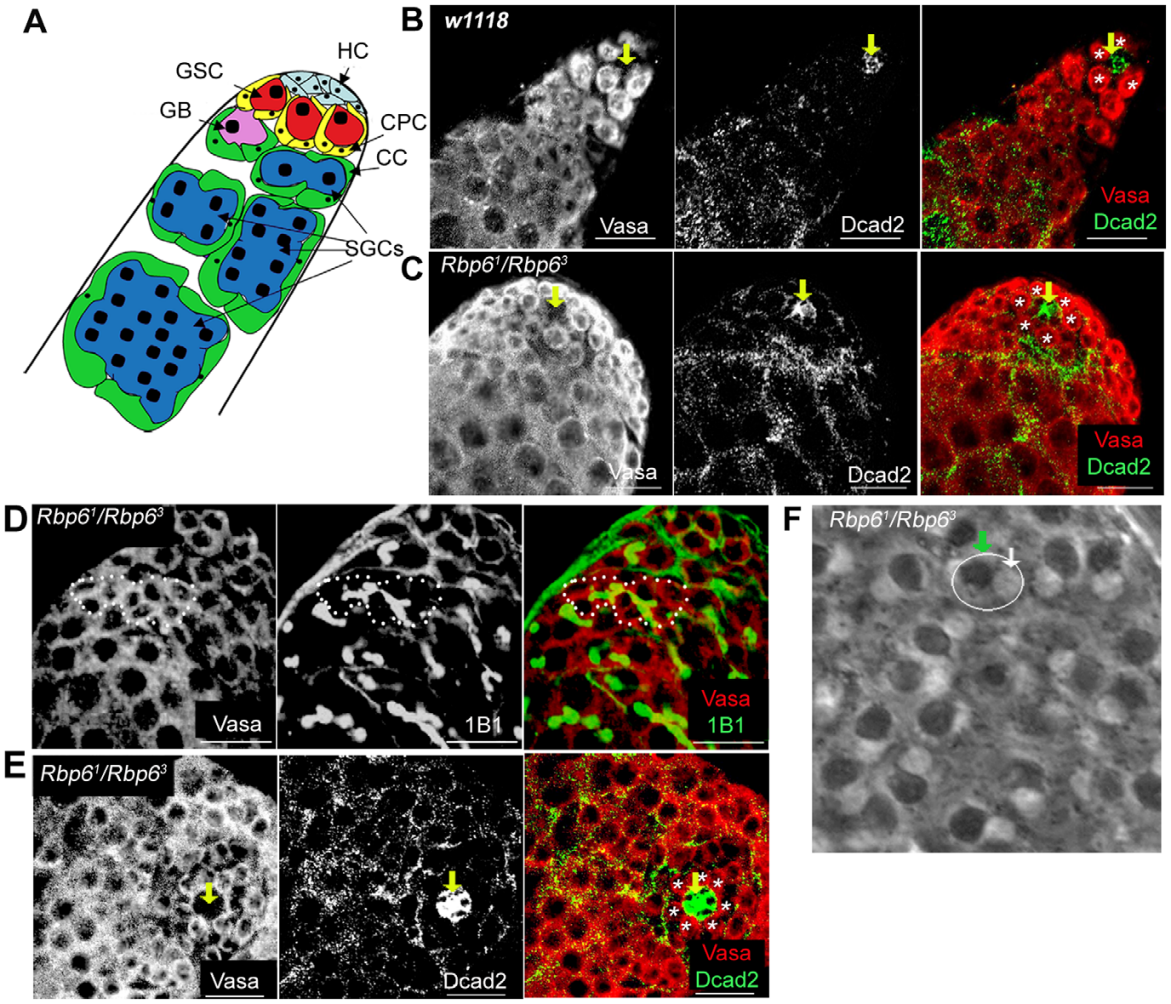
Rbp6 is not required for spermatogenesis. (A) Cartoon representation of the apical region of the *Drosophila* testis. GSCs (red) are located next to a group of somatic hub cells (HC, pale blue). Each GSC is surrounded by a somatic stem cell called a cyst progenitor cell (CPC, yellow). The GSC daughter, called the gonialblast (GB, pink), undergoes four rounds of mitosis to generate a cyst of 16 interconnected spermatogonial germ cells (SGCs, dark blue) (adapted from [10]). (B) Confocal image of a *w^1118^* adult testis stained with a germ cell marker (Vasa, red) and a marker that accumulates on somatic cell membranes (Dcad2, green). GSCs are labelled (*) in the merged image, hub is labelled with an arrow. (C) *Rbp6^1^/Rbp6^3^* mutant testis have normal numbers of Vasa-labelled GSCs (*) surrounding the hub (arrow). (D). A cyst of 8 interconnected germ cells (Vasa, red, white dotted line) connected by the spectrin-rich fusome (1B1 (adducin-related), green) in a *Rbp6^1^/Rbp6^3^* mutant testis. Cysts appear to be generated normally in *Rbp6* mutant testes. (E) A *Rbp6^1^/Rbp6^3^* mutant testis with a displaced hub (Dcad2, green) have normal numbers of GSCs (*) abutting the hub (arrow). (F) A phase contrast micrograph of onion-stage spermatids in a *Rbp6^1^/Rbp6^3^* testis shows one haploid nucleus (white arrow) and one mitochondrial derivative (green arrow), each approximately the same diameter. Scale bars: 20 µm.

### Rbp6 Antibody Analysis Reveals Tissue-specific Expression of Rbp6 throughout Development, but *Rbp6* Deletion Mutants are Viable and Fertile

A Rbp6-specific rat polyclonal antibody was generated to analyse Rbp6 protein expression throughout development. The antigenic peptide sequence was designed to the C-terminus of isoform A. A significant portion of this peptide sequence is also shared by isoforms C, D, E and F making expression of these shorter splice isoforms also detectable with the antibody (Fig. 4A). Isoform B only shares 10 amino acids with the antigenic peptide sequence, making detection of this isoform unlikely.

**Figure 7:**
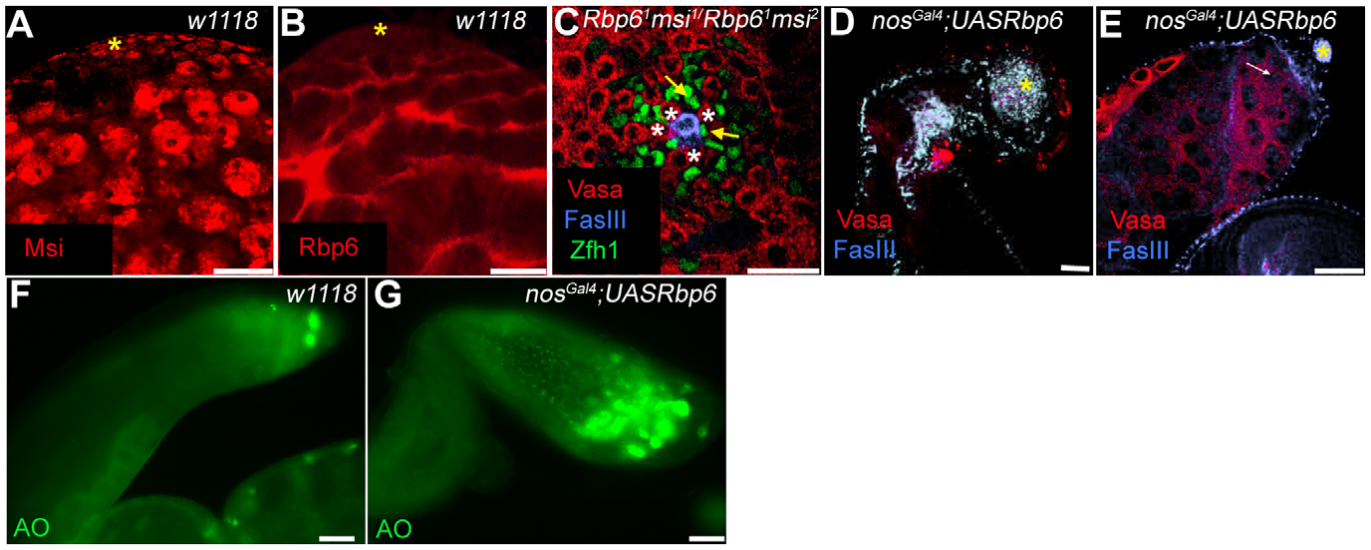
Rbp6 and Msi do not function co-operatively to regulate GSC identity, but mis-expression of Rbp6 causes loss of germ cells through apoptosis. (A) Msi expression (red) in a *w^1118^* third instar larval testis is nuclear and found in germ cells of all stages. (*) denotes the hub. (B) Rbp6 antibody expression (red) is cytoplasmically localised to the cyst cells of the *w^1118^* third instar larval testis. (C) A representative confocal micrograph of a *Rbp6^1^,msi^1^/Rbp6^2^,msi^2^* 2 day old adult double mutant testis containing germ cells (red) of varying stages, from GSCs (*) to spermatocytes. CPCs (Zfh1, green) abut the hub (FasIII, blue) and take over positions where GSC’s have been lost (arrows) as a result of the *msi* mutation. No enhancement of the *msi* mutant phenotype was observed. (D–E) *nos^Gal4^*;*UAS-Rbp6* mutant testes vary from containing no germ cells (red, D) to containing only large spermatocytes in close proximity to the hub (white arrow, E). Acridine Orange labelling of *w^1118^* testes (green, F) and *nosGal4*;*UAS-Rbp6* mutant testes (green, G) reveal more cell death in mutant tissue. Scale bars: 20 µm.

To validate the specificity of the antibody and analyse the function of Rbp6 in development, we constructed deletion mutants using Flp-mediated recombination between pairs of piggyBac transposons carrying FRT sites available from the DrosDel Harvard Exelexis stock collection [31]. We recovered deletions covering the 5′ region of *Rbp6* (*Rbp6^2^*) leaving the four short splice forms intact, the 3′ region of *Rbp6 (Rbp6^1^*) which would presumably cause a complete loss of function of the gene, and a deletion that spans the entire gene (*Rbp6^3^*; Fig 4A). The *Rbp6^1^* and *Rbp6^2^* deletions could be selected on the basis of a change in eye colour following the recombination event and the deletions were confirmed by PCR (not shown). The *Rbp6^3^* full length deletion mutation was confirmed by PCR (not shown) and antibody analysis (see Fig. 4E). To test the specificity of the antibody and validate the genetic nature of the deletion mutants, immunofluorescence and confocal microscopy analysis was performed on wild- type and mutant *Drosophila* tissue at different developmental stages. In wild-type tissue, expression of Rbp6 was detected in the neuronal cells of third instar eye discs, in the cytoplasm of somatic cells of the adult testis, in the third instar ring gland, in the non-proliferating cells of the larval brain lobe, and in the oocyte and cytoplasm of nurse cells in the adult ovary (Fig. 4B–B’’’’’). In *Rbp6^2^* homozygous mutants, expression of Rbp6 was lost from the ring gland and ovary, but was still present in the eye, brain and testis (Fig. 4C–C’’’’’), suggesting that Rbp6 isoform A is the predominant isoform expressed in the ring gland and ovary, while the shorter forms are expressed in the other tissues. We therefore analysed antibody expression in homozygous *Rbp6^1^* and *Rbp6^3^* mutant tissue. No antibody expression was detected in eye discs, larval brains or adult testes dissected from either of these homozygous mutants (Fig. 4D–E’’). Antibody expression was also not detected in the ovary or ring gland of these mutants (not shown). These results validate both the specificity of the Rbp6 polyclonal antibody and the genetic nature of the *Rbp6* deletion mutants.

It was surprising that all *Rbp6* homozygous mutants generated in this study were viable, and adults showed no obvious external morphological defects (not shown). *Rbp6^3^* and *Rbp6^1^* homozygous mutants were fertile (not shown). When trans-heterozygote *Rbp6^3^/Rbp6^1^* mutants were crossed to a 3^rd^ chromosome deficiency covering the region, mutants were slightly slower to eclose than their balanced heterozygote counterparts in a populated vial, indicative that Rbp6 mutants exhibit a slight developmental delay (Fig. S1). *Rbp6^2^* homozygous mutants were male sterile, but we found this to be due to a second site mutation from the parental line used to generate the mutant. *Rbp6^2^* mutants crossed to a 3^rd^ chromosome deficiency covering the region were viable and fertile, as were *Rbp6^3^/Rbp6^1^*, *Rbp6^3^/Rbp6^2^* and *Rbp6^2^/Rbp6^1^* mutants (not shown).

### Rbp6 is Not Widely Expressed in *Drosophila* Stem Cell Populations

Since vertebrate Msi is widely used as a stem cell marker, we also examined whether Rbp6 might be expressed in other *Drosophila* stem cell populations, including intestinal stem cells, hematopoietic stem cells, larval neuroblasts and somatic or germline stem cells of the ovary. Rbp6 antibody expression was not detectable in the lymph gland (the source of hematopoietic stem cells), intestinal stem cells, or dividing neuroblasts of the third instar brain (Fig. 5A–C). We did observe weak antibody expression in the germarium of the *Drosophila* ovary where germline and somatic stem cells reside (Fig. 5D). However, *Rbp6^1^/Rbp6^3^* mutant ovaries (N = 50) were phenotypically normal (not shown) and exhibited no morphological defects, indicating that Rbp6 is not required for the regulation of ovarian stem cell populations.

### Numb is not a Target of Rbp6

Numb, a negative regulator of Notch signalling, has been reported to be a target of vertebrate Msi2 in hematopoietic cells [39] and Msi1 can repress Numb in NIH3T3 cells [40]. We addressed whether *Drosophila numb* mRNA could be a potential target of Rbp6 in the third instar larval brain and the adult testis, two tissues in which Rbp6 is expressed. Since Msi proteins target mRNA and prevent translation of the protein product, we would expect an increase in Numb protein expression in loss of function mutant tissue. However, we observed no obvious change in Numb antibody expression levels or distribution in *Rbp6^1^/Rbp6^3^* mutant tissue when compared to wild-type tissue, revealing that Numb is an unlikely target of Rbp6 (Fig. S2).

### Rbp6 Function is not Required for *Drosophila* Spermatogenesis

Since *Drosophila msi* is required for the maintenance of male germline stem cell (GSC) identity and for male meiotic chromosome segregation and cytokinesis (Siddall et al., 2006), we sought to determine whether Rbp6 played a similar role in male spermatogenesis. In the *Drosophila* testis, approximately 5–9 GSCs cluster around a group of non-dividing somatic cells called hub cells [41]. Hub cells are localized to the apical tip of the testis and remain tethered to the basal lamina. GSCs, which are attached to the hub cells, divide asymmetrically to produce a daughter gonialblast (GB), which then undergoes four rounds of mitotic divisions with incomplete cytokinesis to produce a cyst of sixteen interconnected spermatogonial cells, the precursors to sperm (Fig. 6A) [42]. To determine whether Rbp6 function is required in the regulation of spermatogonial GSC behaviour, we dissected and stained testes with cell specific markers from *Drosophila Rbp6^1^/Rbp6^3^* transheterozygote mutant adults to control for any alternative site mutations that may be present from the parental lines used to generate the deletions. Males of this genotype were fertile, and the testes analysed had an average of 8.8±.22 (± SEM) stem cells per testis (N = 20), as determined by the number of Vasa-positive germ cells attached to the DE-Cadherin (Dcad2) labelled hub (somatic niche) cells. This number was not significantly different from the average number of stem cells we observed in *w^1118^* testes (8.47±.43, N = 15, p>.05; Fig. 6B–C). Furthermore, we observed no abnormalities in the number of germ cells present in germline cyts, with each cyst containing 2, 4, 8 or 16 germ cells connected by a spectrin-rich fusome, which remains intact throughout most of spermatogenesis [43] (Fig. 6D). The only defect we observed was the occasional mis-localisation of the somatic hub in 5 out of 20 testes analysed (Fig. 6E). In each of these cases, a rosette of Vasa-expressing stem cells was observed around the irregularly localised hub cells (Fig. 6E), indicating that while the hub was no longer attached to the basal lamina, no loss of stem cells was ever observed.

To test for meiotic defects, we dissected mutant testes and examined live preparations under phase contrast microscopy. In mutant testes, haploid early round spermatids appeared normal, containing a single nucleus and mitochondrial derivative of approximately equal size (Fig. 6F). In no case did we ever observe more than one nucleus associated with an abnormally large mitochondrial derivative, which is suggestive of meiotic problems and is a phenotype associated with *msi* mutants (Siddall et al; 2006). Together, these results lead us to conclude that Rbp6 is not required for maintenance of germline stem cells or for male meiosis. Thus Rbp6 and its paralog Msi do not have similar roles in *Drosophila* spermatogenesis.

### Rbp6 and Msi do not Function Co-operatively to Regulate Spermatogenic Germline Stem Cell Identity

Since functional analysis of Msi-1 and Msi-2 in mammalian NSC regulation has revealed the co-operative nature of these two proteins in maintaining the undifferentiated state of NSCs [15], we considered the possibility that *Drosophila* Rbp6 and Msi may function redundantly in the regulation of spermatogenic stem cells. Our previous detailed characterisation of Msi expression revealed that Msi was expressed in the nucleus of all somatic cells and germ cells, including GSCs, of the testis [10] (Fig. 7A). Despite the fact that we only detected Rbp6 expression in the cytoplasm of cyst cells in the testis (Fig. 4B’; 7B), we could not rule out the possibility that Rbp6 expression may be undetectable in the germline using fluorescent microscopy techniques, or that Rbp6 isoform B, which is not detectable with the antibody we generated, may be expressed in germ cells. *Rbp6^1^msi^1^/Rbp6^1^msi^2^* mutant recombinant flies were generated and adult testes were dissected and labelled with specific germ cell and somatic cell markers to visualise stem cells. We have previously documented a mild to moderate loss of GSCs in *msi* mutant pharate adult testes [10]. We analysed *Rbp6^1^msi^1^/Rbp6^1^msi^2^* mutant adult testes at 2 days post eclosion to determine whether there was any enhancement of the *msi* single mutant phenotype, and found that 96.6% of testes (N = 30) retained at least four GSCs abutting the hub (see Fig. 7C for a representative image). Furthermore, 100% of testes contained differentiated germ cell progeny. The double mutant testes resembled that of a single *msi* mutant [10], with a mild loss of GSCs evident by the replacement of some GSCs with somatic stem cells (SSCs) marked with the SSC-specific marker, Zfh1 [44] (Fig. 7C). Additionally, the majority of testes exhibited a swelling of the apical region due to defects in the development of the outer sheath, a phenotype also attributable to *msi* mutations [10]. These results suggest that Rbp6 and Msi do not function redundantly to maintain the spermatogenic stem cell fate. Interestingly, *Rbp6^1^msi^1^/Rbp6^1^msi^2^* mutant recombinant flies were viable for approximately 5 days post eclosion, and adults phenotypically resembled *msi^1^/msi^2^* mutants (not shown), further suggesting that Rbp6 and Msi share no functional redundancy in *Drosophila* development.

### Rbp6 Mis-expression in *Drosophila* Germ Cells Causes Germ Cell Loss Due to Cell Death

*Rbp6* mutants are viable and fertile and do not show any obvious phenotypic defects. Although Msi is required in the *Drosophila* germline to regulate germline stem cell behaviour [10], Rbp6 is not expressed in male germ cells and none of the loss of function mutants generated in this study revealed any spermatogenic defects (Fig. 6). We therefore asked whether forced mis-expression of Rbp6 would have any functional consequences, and to this end, we used the testis as a model. To determine the consequences of mis-expressing Rbp6 in *Drosophila* testis germ cells, we generated a UAS-Rbp6 construct. The construct was driven in early germ cells using a *nanos* ^Gal4^ (*nos^Gal4^*) driver [30]. Over half the *nos^Gal4^;UAS-Rbp6* 3-day old adult testes (N = 12) analysed had no Vasa-positive germ cells remaining in the testis (Fig. 7D), while the remainder had large Vasa-positive spermatocytes abutting the somatic hub (Fig. 7E). Thus mis-expression of Rbp6 in germ cells caused germ cells to be lost from the testis. We examined whether these cells were being lost from the testis due to cell death by comparing acridine orange (AO) distribution and levels in *w^1118^* testes and *nosGal4;UAS-Rbp6.* We observed significantly more AO-positive cells in *nosGal4;UAS-Rbp6* testes than in *w^1118^* tissue (Fig. 7F–G), indicating that germ cells are being lost due to cell death rather than premature differentiation. Thus regulation of Rbp6, at least in germ cells, is important since mis-expression has the functional consequence of germ cell loss due to apoptosis.

## Discussion

The highly conserved Msi family of RNA-binding proteins have members present in a number of different organisms, ranging from primitive placozoans to higher vertebrates. Msi was first discovered in *Drosophila*, with the vertebrate orthologues, Msi-1 and Msi-2, subsequently identified. These proteins have attracted much interest due to their expression in a number of different vertebrate stem cell populations, including neural, epithelial and germline stem cells [5], [10], [15], [17], [22]. Furthermore, an increase in Msi-1 expression has been associated with various tumours of both epithelial and neural origin, some of which are thought to have a stem cell origin [23], [25], [26], [45], [46], [47]. In addition, important recent research has shown that Msi-2 plays a role in the regulation of hematopoietic stem cells, and increased expression can promote leukemic progression [48]. Since Msi expression is associated with stem/progenitor populations of cells, it is interesting that a Msi protein sequence is present in the placozoan, *Trichoplax adhaerens,* which is a simple, marine living invertebrate consisting of just four cell types [49]. However, one study has suggested that a multipotent population of peripheral cells contributes to the epithelial boundary of *T. adhaerens* [50], reflecting the possibility that Msi family members may have a function in stem-like cells in the simplest of organisms.

In vertebrates, the expression patterns of Msi-1 and Msi-2 have diverged in a number of tissues, including the subventicular and venticular zone of the mouse brain [14], in the mouse testis [10], and in intestinal epithelial cells [17]. In each of these cases, Msi-2 expression appears to be more broadly distributed in cells of these tissues, and overlapping expression of Msi-1 and Msi-2 is not always apparent. Thus while Msi proteins can compensate for each other’s function, these protein family members have also evolved separate functions in vertebrates.

We previously showed *d*Msi to be a key regulator of at least one adult stem cell population in *Drosophila* [10] and in this way it shares similar functions with its vertebrate orthologues. In our current study, we describe the identification of a second *Drosophila* Msi family member, Rbp6, which we show to be the most similar in amino acid identity to vertebrate Msi family members. Although Rbp6 is expressed in a distinct manner in multiple tissues throughout *Drosophila* development, we were surprised that the generation of *Rbp6* deletion mutants revealed no function for this gene, with adults being viable and fertile. The only mutant phenotype we did observe was the occasional mis-localization of the niche hub cells in the adult testes of a small proportion of *Rbp6* mutant transheterozygotes. Thus it appears that Rbp6 is dispensable for the normal development of the fly, which is surprising, since the sequence conservation between Rbp6 and its vertebrate orthologues suggests selection for this gene. Despite our inability to demonstrate a functional relatedness of Rbp6 to Msi, sequence comparison clearly shows that Msi and Rbp6 have evolved from a common ancestor and that Rbp6 has an overall higher sequence similarity to vertebrate family members than Msi. Alterations to regulatory elements and specific amino acids in Rbp6 may have lead to a divergence of expression pattern and function from other Msi family members. It is possible in our study that we have failed to uncover some more subtle phenotypes associated with *Rbp6* mutations, such as a behavioural phenotype or phenotypes only uncovered under conditions of stress. There are other examples of genes conserved between invertebrates and vertebrates where a clear role for the vertebrate gene is defined, but a function for the invertebrate gene remains elusive [51], [52].

Using the adult testis as a model, we tested whether Rbp6 and Msi may share some functional redundancy in the regulation of an adult stem cell population, such as is the case in vertebrate neural stem cells with Msi-1 and Msi-2 [15]. However, the *msi* mutant testis phenotype was not exacerbated in *Rbp6 msi* double mutants. Furthermore, *Rbp6 msi* double mutants were viable and appeared phenotypically similar to *msi* single mutants (not shown) suggesting no overlap in function between *d*Msi and Rbp6. Since Rbp6 shares some sequence homology to a number of RRM-containing proteins in *Drosophila*, including Msi, it is possible that Rbp6 may share some functional redundancy with other RNA-binding proteins with similar RRM domains.

Finally, we provide evidence that forced mis-expression of Rbp6 leads to a loss of germ cells in the testis by cell death. This suggests that Rbp6 levels needs to be tightly regulated, and gives at least some evidence of the functionality of Rbp6. The role of Rbp6 in *Drosophila* development, however, has clearly diverged from its paralogue, *d*Msi, the latter being more biological relevant in stem cell biology and cell cycle, like its vertebrate orthologues.

## Supporting Information

Figure S1 ***Rbp6*** **transheterozygote mutants are slower to eclose in a competition setting than their balanced heterozygote counterparts.** Graph plotting the percentage of *Rbp6* mutants (blue line) vs balanced heterozygotes (black line) from 11 days after egg deposition (AED). The *Rbp6* mutant genotypes were either *Rbp6* ^3^/*Df(3L)81k19* or *Rbp6* ^1^/*Df(3L)81k19* and the balanced heterozygote genotype was either *Rbp6* ^3^ */TM3*-GFP or *Rbp6* ^1^ */TM3*-GFP. The majority of balanced heterozygotes eclose by 12 days post AED. Eclosion of *Rbp6* mutants appears to be delayed by 24 hours.
(TIF)

Figure S2 **Numb expression is not altered in Rbp6 mutant tissue.** (A–D). Numb expression remains unchanged in *Rbp6^1^/Rbp6^3^* mutant 3^rd^ instar brain lobes (B) compared to wild-type brain lobes (A), and in *Rbp6^1^/Rbp6^3^* mutant adult testes (D) compared to wild-type testes (C). Scale bars: 20 µm.
(TIF)
