# Supplementary figures and images for: Drosophila Rbp6 Is an Orthologue of Vertebrate Msi-1 and Msi-2, but Does Not Function Redundantly with dMsi to Regulate Germline Stem Cell Behaviour
Source: PLoS One. 2012 Nov 27;7(11):e49810. doi: 10.1371/journal.pone.0049810 (PMC3507872; doi:10.1371/journal.pone.0049810)

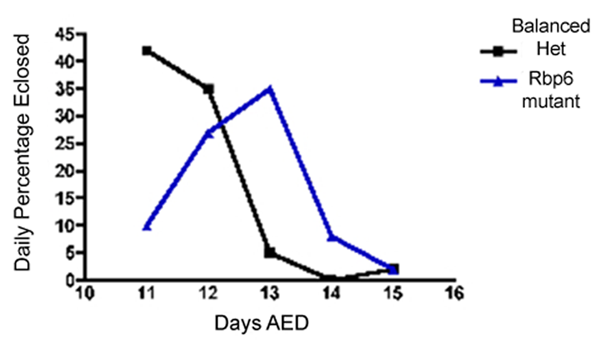

Supplement: Figure S1 — Rbp6 transheterozygote mutants are slower to eclose in a competition setting than their balanced heterozygote counterparts. Graph plotting the percentage of Rbp6 mutants (blue line) vs balanced heterozygotes (black line) from 11 days after egg deposition (AED). The Rbp6 mutant genotypes were either Rbp6 3/Df(3L)81k19 or Rbp6 1/Df(3L)81k19 and the balanced heterozygote genotype was either Rbp6 3 /TM3-GFP or Rbp6 1 /TM3-GFP. The majority of balanced heterozygotes eclose by 12 days post AED. Eclosion of Rbp6 mutants appears to be delayed by 24 hours. (TIF) [file pone.0049810.s001.tif]

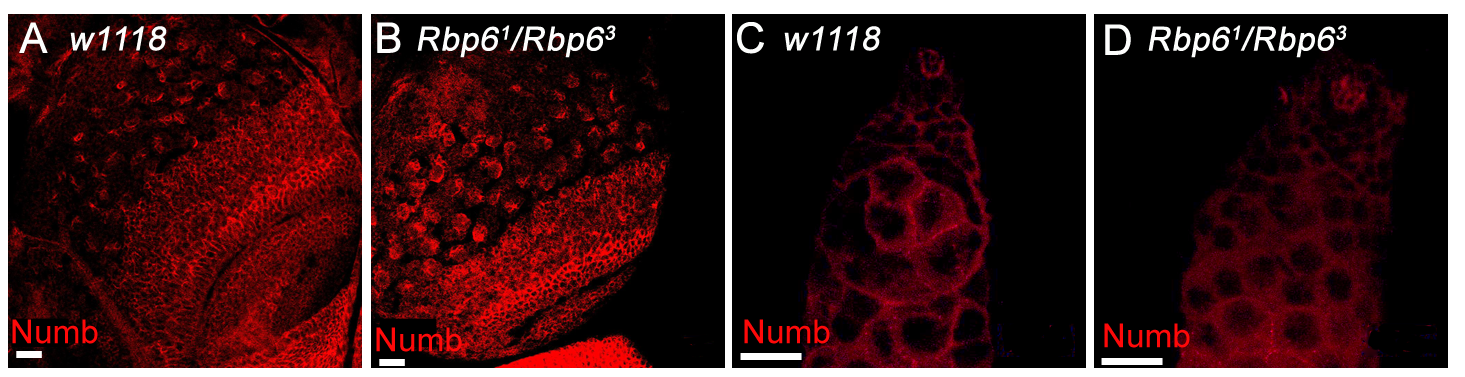

Supplement: Figure S2 — Numb expression is not altered in Rbp6 mutant tissue. (A–D). Numb expression remains unchanged in Rbp61/Rbp63 mutant 3rd instar brain lobes (B) compared to wild-type brain lobes (A), and in Rbp61/Rbp63 mutant adult testes (D) compared to wild-type testes (C). Scale bars: 20 µm. (TIF) [file pone.0049810.s002.tif]
